# Systematic review on the needle and suture types for uterine compression sutures: a literature review

**DOI:** 10.1186/s12893-019-0660-z

**Published:** 2019-12-16

**Authors:** Shinya Matsuzaki, Mariko Jitsumori, Takeya Hara, Satoko Matsuzaki, Satoshi Nakagawa, Tatsuya Miyake, Tsuyoshi Takiuchi, Aiko Kakigano, Eiji Kobayashi, Takuji Tomimatsu, Tadashi Kimura

**Affiliations:** 1 Department of Obstetrics and Gynecology, Osaka University Graduate School of Medicine, 2-2 Yamadaoka, Suita, Osaka, 565-0871 Japan; 2 Department of Obstetrics and Gynecology, Otemae Hospital, Osaka, Japan

**Keywords:** Uterine compression suture, Needle, Suture size

## Abstract

**Background:**

This study aimed to identify and review associations between the types of sutures used for uterine compression suture (UCS) and its outcomes in postpartum hemorrhage.

**Methods:**

An electronic search using PubMed and Scopus databases was performed. We included the English articles reported from January 1, 1997, to May 31, 2017, using search words or terms regarding the types of suture and needle used for UCSs. We only included studies describing the sutures in the systematic review.

**Results:**

We found 196 studies and included 76 (38.8%) in our analysis. We collected data on maternal outcomes for 924 patients and categorized them. Of the 76 studies, suture sizes 0, 1, and 2 were used in 6, 44, and 32 articles, respectively (some studies used multiple sutures). Of the 45 studies mentioning the needles, curved and straight needles were used in 35 and 10, respectively. The results of our review revealed that about 80% of previous articles used Catgut and Polyglactin 910 sutures. Because no studies that compared the efficacy of different size of sutures were identified, we investigated the differences using the cases reported in previous studies mentioned above. In the first analysis, we compared the uterine preservation rate between size 1 and size 2 sutures. We found no significant difference in uterine preservation rate (92.8%: size 1 vs. 94.2%: size 2, *p* > 0.05) but found significant difference in transfusion rate (62.4% vs. 79.1%, *p* < 0.01). With the hypothesis that non-transfusion cases were less severe, we excluded these cases from second analysis. Although our second analysis of only Catgut or Polyglactin showed strong selection bias, we observed that uterine preservation rate was significantly higher in cases with size 2 suture than in those with size 1 suture (86.9% vs. 93.5%, *p* = 0.033).

**Conclusions:**

Our systematic review showed that approximately 80% of cases were treated by Catgut and Polyglactin 910. Due to the heterogeneity of cases included in this review, it is difficult to estimate which suture is better for UCSs. More robust studies are necessary to enable the identification of the superior suture for performing UCSs.

## Background

Severe hemorrhage after birth is known as postpartum hemorrhage (PPH), and it is a major reason for maternal death worldwide, with an incidence in 1–2% of live births. Uterine compression sutures (UCSs) have been indicated to manage PPH [1–3]. Reported UCS techniques have shown similar success rates (76–100%) for uterine preservation [4–14], and these reports have proven that UCSs are essential treatment options for PPH.

Although many UCS techniques are considered valuable, differences in the efficacies of different needles and sutures have not been well studied as most studies have focused on modification of the UCS procedure and in turn disregarded the influence of the choice of needles and sutures used. Thus, we focused on the efficacies of needles and sutures used for UCSs to identify the best ones for UCSs and help improve patient outcomes. In our previous study, we performed literature search on suture and needle types used for UCSs; however, we only investigated the results on dedicated needles for UCSs [15].

Here, we present the results of our systematic literature review to compare the efficacy of different types of needle and suture used for UCSs.

## Methods

### Factors

We conducted a systematic search of articles published between January 1, 1997, and May 31, 2017, using PubMed and Scopus databases, as performed in our previous study [15, 16]. We included only English articles and excluded articles reported before 1997. The search strategy involved the use of these keywords: uterine compression suture, B-Lynch, and uterine preservation surgery. Articles on B-Lynch sutures were also included.

### Article retrieval

We reviewed the retrieved articles following the Preferred Reporting Items for Systematic Reviews and Meta-Analyses guidelines [17]. Two authors (SM and MJ) independently performed the study selection (screening of titles, abstracts, and full texts of relevant articles) as described [18]. Many articles we found had insufficient information regarding the needles; thus, we chose all those that included information on sutures with or without needles for UCSs (suture sizes were those defined by the United States Pharmacopeia). We excluded articles that did not mention the suture type or uterine preservation rate. The outcomes and complication rates for each suture type were not available in all articles.

Given the reported similarity in high uterine preservation rates among the types of UCS [4], we assumed equivalent efficacies among UCS techniques in our analysis.

As mentioned in our previous study, our institution standardized the timing for performing UCSs to avoid performing unnecessary sutures [6]. However, most publications have failed to mention UCS timings, and these certainly vary among facilities; thus, comparing the different needle and suture types to determine the efficacy of UCSs was challenging. We limited our analysis to transfused cases and to those using Polyglactin 910 or Catgut sutures for maximum alignment, to compare the uterine preservation rates between suture sizes 1 and 2 in studies with similar conditions. Thus, we assumed that all patients who underwent hysterectomy had received transfusions.

Our primary aim with this study was to investigate the efficacies of UCSs with different needle and suture types. Our secondary aim was to reveal the needle and suture types used in previous studies.

### Statistical analysis

Statistical analyses were performed using JMP Pro (version 13.1; SAS Institute, Tokyo, Japan). Categorical variables were analyzed using χ^2^ and Fisher’s tests and continuous variables using one-way analysis of variance. *P*-value < 0.05 was considered to indicate statistical significance.

## Results

### Search results

**Fig. 1:**
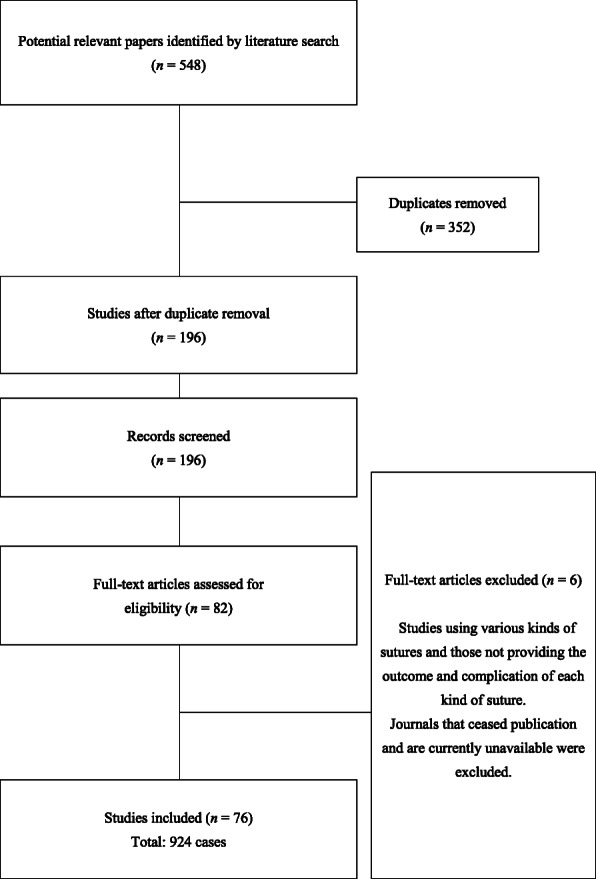
Flow diagram of study selection for the systematic review

Figure 1 presents a flow diagram of the literature search. Of the 196 nonduplicate records identified from the electronic database search and other sources, we retrieved 196 full-text articles after screening the titles and abstracts. After excluding the articles that did not meet the inclusion criteria (56 case series and 20 case reports), we included 76 articles for analysis. Additional file 1: Table S1 and Additional file 2: Table S2 provide detailed information and references of all included articles.

### Results of the literature review on the type of needles and sutures used for UCS

All 76 articles described UCSs with suture size and 45 included needle types. Of the 76 studies, suture sizes 0, 1, and 2 were used in 6, 44, and 32 articles, respectively (some studies included procedures with different suture and needle types; Table 1). Of the 45 studies that included needle types, 35 used curved needles and 10 straight needles. Sutures size 1 and curved needles were used most often. Determining the association between the procedure efficacy and the needle types was difficult because various types of needles were used during the UCSs; thus, we focused on the differences among suture types. The results of our review showed that about 80% of the studies included used Catgut or Polyglactin 910 sutures (19 and 45 studies, respectively).

**Table 1:** Summary of published results on the review of the type of sutures used for uterine compression sutures

Suture size	Suture material	Number or studiesa	Total cases included in the studies		Needle types		
					Curved	Straight	NA
No. 0	Polyglactin 910	4	13	Shape	3	0	0
				Bodied	0	0	0
				NA	0	0	1
	Catgut	1	5		NA		
	Poliglecaprone 25	1	1	NA	0	1	0
No. 1	Polyglactin 910	25	355	Shape	3	0	0
				Bodied	5	1	0
				NA	6	1	9
	Catgut	7	85	Shape	1	1	0
				Bodied	2	0	0
				NA	0	1	2
	Polydioxanone	2	2		NA		
	Poliglecaprone 25	3	36	Bodied	1	0	0
				NA	0	0	2
	Others	7	98	Shape	2	1	0
				Bodied	1	0	0
				NA	0	0	3
No. 2	Polyglactin 910	16	128	Shape	0	0	0
				Bodied	2	0	0
				NA	3	2	9
	Catgut	11	155	Shape	0	0	0
				Bodied	3	0	0
				NA	2	1	5
	Polydioxanone	1	1		NA		
	Others	4	25	Shape	0	0	0
				Bodied	1	0	0
				NA	0	1	2

^a^Some studies overlapped

### Transfusion rate

The transfusion rates ranged from 0 to 100% and varied between studies, with 42 studies mentioning about transfusion rate (including ≥5 cases of UCS). Of the 42 studies, 26 (61.9%) showed more than 80% transfusion rate, 13 (31.0%) showed 10–79%, and 3 (7.1%) showed 0–9%.

### Comparison of the UCS surgical outcomes for different suture sizes

Table 2 shows the surgical outcomes for each suture size. A significantly lower transfusion rate was observed in the group of UCSs performed with size 1 sutures than in the group of UCSs performed with size 2 sutures (62.4% vs. 79.1%, respectively, *p <* 0.01). However, the uterine preservation rates did not significantly differ between the groups (92.8% vs. 94.2%, *p =* 0.249). Four uterine necroses (0.67% of the total 596 UCSs) occurred in procedures performed with size 1 sutures, and none occurred in those performed with size 2 sutures. As shown in Table 2, we found no significant differences in the rates of severe complications (complications needing surgical interventions) after procedures performed with either size 1 or size 2 sutures (*p =* 0.332).

**Table 2:** Review of the suture types used for uterine compression sutures in previous studies (all cases)

Suture size	Suture material	Total cases included in the studies	Estimated mean blood loss (ml)	Transfusion rate	Uterine preservation rate	Severe complication rate^a^
No. 0	Polyglactin 910	13	3082	9/10 (90%)	13/13 (100%)	0
	Catgut	5	2830	5/5 (100%)	5/5 (100%)	0
No. 1	Polyglactin 910	375	1921	211/372 (56.7%)	342/375 (91.2%)	3/375 (0.8%)
	Catgut	85	1380	34/40 (85.0%)	82/83 (98.8%)^b^	1/83 (1.2%) ^b^
	Others	136	2309	97/136 (71.3%)	127/136 (93.4%)	1/136(0.74%)
	Total	596	2039	342/548 (62.4%)	551/594 (92.8) ^b^	5/594 (0.91%) ^b^
No. 2	Polyglactin 910	128	2135	92/102 (90.2%)	120/128 (93.8%)	0/128 (0%)
	Catgut	155	2361	78/118 (66.1%)	146/155 (94.2%)	0/124 (0%)
	Others	26	2531	19/19 (100%)	25/26 (96.2%)	0/19 (0%)
	Total	309	2249	189/239 (79.1%)	291/309 (94.2%)	0/271 (0%)

^a^Severe complications are defined as the need for surgical intervention to treat complications due to uterine compression sutures

^b^Two maternal deaths due to the hypertention disorder of pregnancy were excluded

We performed second analysis by limiting our analyses to cases requiring transfusions with the hypothesis that non-transfusion cases were less severe. We only included the cases using Polyglactin 910 or Catgut sutures in this analysis. The uterine preservation rate was significantly higher in the procedures performed with size 2 sutures than in those performed with size 1 sutures (Table 3; 86.9% vs. 93.5%, respectively, *p =* 0.033). Next, we compared the outcomes after size 1 and 2 suture UCSs (Polyglactin 910 and catgut) in cases of uterine atony. There was no significant difference in the rates of transfusion and uterine preservation in the analysis of limited transfusion cases of uterine atony (89.3% vs. 92.5%, *p* = 0.428) (Table 3 and Additional file 3: Table S3).

**Table 3:** Comparison of uterine preservation rate among different suture sizes

Suture size	Suture material	Total cases included in the studies	Mean blood loss (ml)	Transfusion rate	Uterine preservation	Severe complication rate^a^
No. 1	Total	594^b^	2039	**342/548(62.4%)**^**c**^	551/594 (92.8%)^d^	5/594 (0.84%)^e^
	Limited transfusion cases (Polyglactin 910 and catgut)				**213/245 (86.9%)**^**f**^	
	Limited transfusion and uterine atony cases (Polyglactin 910 and catgut)				142/159 (89.3%)^g^	
No. 2	Total	309	2249	**189/239 (79.1%)**^**c**^	291/309 (94.2%)^d^	0/309(0%)^e^
	Limited transfusion cases (Polyglactin 910 and catgut)				**159/170 (93.5%)**^**f**^	
	Limited transfusion and uterine atony cases (Polyglactin 910 and catgut)				135/146 (92.5%)^g^	

Bold indicates statistical significance

^a^Severe complications are defined as need for surgical intervention to treat complications due to uterine compression sutures

^b^Two maternal deaths due to the hypertention disorder of pregnancy were excluded

Statistical analysis was performed among b, c, d, and e. The *p* values are listed below

^c^*p* = 0.000

^d^*p* = 0.249

^e^*p* = 0.332

^f^*p* = 0.033

^g^*p* = 0.428

## Discussion

The key finding of our study was that approximately 80% cases were treated by Catgut and Polyglactin 910 sutures with size 1 or 2 for UCSs. This systematic review is the first to compare different sutures (sizes 1 or 2) used for UCSs. However, we conclude that it is difficult to estimate which suture is better for UCSs due to the heterogeneity of cases included in this review.

Our review also found that transfusion rates varied from 0 to 100% and more than half of the studies showed high transfusion rate (≥80%). First, UCS was indicated only in severe hemorrhage cases [5]. However, recently, UCS is used in mild cases, even though “prophylactic UCS” is currently performed [19]. We considered that these differences might reflect the differences in the initiation times of UCSs in each study.

We compared different types of sutures used for UCSs; however, the analysis was difficult because when performing UCSs for mild cases, the transfusion rate was low and the uterine preservation rate may have been high with the use of UCSs. For example, El Refaeey AEA et al investigated 108 cases [20], which included the largest number of UCSs in this review, and reported the rates of transfusion and uterine preservation of only 9.3% (10 of 108) and 100% (108/108) cases, respectively. The authors performed “early” intervention, resulting in the treatment of less severe cases and reduction of the rate of transfusion.

According to our results, the transfusion rate in UCS cases performed with size 1 sutures was significantly lower than that in cases performed with size 2 sutures (62.4% vs. 79.1%, respectively, *p* < 0.01), but the uterine preservation rates were similar between the same groups (92.8% vs. 94.2%, respectively, *p =* 0.249). Therefore, we attempted to limit the analysis of uterine preservation rates to transfused cases and to only analyze cases using Polyglactin 910 or Catgut sutures to reduce the difference in severity between the cases using size 1 and 2 sutures. Although this analysis showed a strong bias, sub-analysis showed that the uterine preservation rate in transfused cases was higher when performed with size 2 sutures than with size 1 sutures (86.9%: size 1 vs. 93.5%: size 2, *p* = 0.033). Moreover, there were no between-group differences in terms of complication rate.

Although our systematic review failed to investigate differences in terms of efficacy among needle types, we found that three dedicated needles have already been reported: poliglecaprone 25, No.1 suture made by Ethicon Endo-Surgery (W3709) for B-Lynch technique [21, 22]; a curved needle with customized sizable, reported by Yano et al. for MY suture [23]; and a straight super blunt needle reported by Matsuzaki et al. [15] These studies failed to fulfill our criteria and were excluded. Whether dedicated needles can improve the uterine preservation rate remains unclear.

The strength of our study lies in it being the first review to compare the efficacies of different needles and sutures for UCSs. Although it was difficult to conclude the superiority of needle and suture for UCS, our data may be useful to investigate the efficacy of needles and sutures in future studies. Second, our results also provide useful information, such as the needle and suture types being used for UCS as well as the transfusion rates of previous studies.

We are aware of our study’s limitations. First, because we did not investigate the efficacy of different UCSs, the efficacy of different sutures was analyzed under the assumption that UCS techniques are equally effective and show the same uterine preservation rate. This assumption may have strongly biased the result of our analysis. Second, we could not investigate the efficacies of different sutures under the same conditions because PPH often occurs because of different causes and because UCS timing varies according to clinicians’ judgment. To adjust the severity of conditions, we limited the analysis of uterine preservation rate to cases requiring transfusions; however, this selection may also have caused biases.

Third, we could not classify the PPH cases according to their causes, and we had to individually determine the efficacies of sutures. Moreover, the relationship between uterine preservation rate and different needles or suture was not compared in the same study, negating the feasibility of a needle- or suture-specific discussion. Fourth, we could not investigate or exclude the effect of adjunctive treatments such as uterine artery ligation for UCS; thus, unmeasured bias may exist in the analysis. Fifth, because previous studies lacked detailed information, we could not determine the timing of transfusion. Therefore, these results may be biased because our hypothesis was that non-transfusion cases exhibit less severity.

As mentioned above, our study has strong biases; however, we believe it is an acceptable method. In order to resolve these biases, better study designs, such as case-control and cohort studies, and perhaps even more robust studies, such as randomized control trials, are expected to provide better evidence regarding the needles and sutures used for UCSs.

## Conclusions

A systematic review determining the associations between size 1 and size 2 sutures used for UCS and its outcomes in postpartum hemorrhage showed that approximately 80% of cases were treated by Catgut and Polyglactin 910. We also revealed that it is difficult to investigate the efficacy of UCSs among the different needles and sutures used based on the current knowledge. Comparisons between size 1 and size 2 sutures in more robust studies are necessary to enable the identification of the superior suture for performing UCSs.

## Supplementary information

**Additional file 1: Table S1**. Detailed information of the sutures and needles in published studies

**Additional file 2: Table S2**. References of all publications on uterine compression sutures that satisfied our criteria

**Additional file 3: Table S3** Comparison of uterine preservation rate among different suture sizes in uterine atony cases

## Data Availability

The dataset used and/or analyzed during the current study are available from the corresponding author on reasonable request.

## Abbreviations

PPH: Postpartum hemorrhage
UCS: Uterine compression suture
