# Supplementary material for: Systematic review on the needle and suture types for uterine compression sutures: a literature review
Source: BMC Surg. 2019 Dec 16;19:196. doi: 10.1186/s12893-019-0660-z (PMC6916157; doi:10.1186/s12893-019-0660-z)
Supplement: Supplementary file 2 — Additional file 2: Table S2. References of all publications on uterine compression sutures that satisfied our criteria [file 12893_2019_660_MOESM2_ESM.docx]

**Table S2. References of all previous studies of uterine compression sutures that satisfied our criteria**

**Reference**

1. Takeda J, Kumakiri J, Makino S, Itakura A, Takeda S. Laparoscopic removal of uterine vertical compression sutures. Gynecology and Minimally Invasive Therapy. 2017;6:73-75

2. Conrad LB, Groome LJ, Black DR. Management of Persistent Postpartum Hemorrhage Caused by Inner Myometrial Lacerations. Obstet Gynecol. 2015;126:266-9.

3. Shazly SA, Badee AY, Ali MK. The use of multiple 8 compression suturing as a novel procedure to preserve fertility in patients with placenta accreta: case series. Aust N Z J Obstet Gynaecol. 2012;52:395-9.

4. Makino S, Tanaka T, Yorifuji T, Koshiishi T, Sugimura M, Takeda S. Double vertical compression sutures: A novel conservative approach to managing post-partum haemorrhage due to placenta praevia and atonic bleeding. Aust N Z J Obstet Gynaecol. 2012;52:290-2.

5. Hackethal A, Brueggmann D, Oehmke F, Tinneberg HR, Zygmunt MT, Muenstedt K. Uterine compression U-sutures in primary postpartum hemorrhage after Cesarean section: fertility preservation with a simple and effective technique. Hum Reprod. 2008;23:74-9.

6. Ferguson JE, Bourgeois FJ, Underwood PB. B-Lynch suture for postpartum hemorrhage. Obstet Gynecol. 2000;95:1020-2.

7. El Refaeey AEA, Abdelfattah H, Mosbah A, Gamal AM, Fayla E, Refaie W, et al. Is early intervention using Mansoura-VV uterine compression sutures an effective procedure in the management of primary atonic postpartum hemorrhage? : a prospective study. BMC Pregnancy Childbirth. 2017;17:160.

8. Rauf M, Ebru C, Sevil E, Selim B. Conservative management of post-partum hemorrhage secondary to placenta previa-accreta with hypogastric artery ligation and endo-uterine hemostatic suture. J Obstet Gynaecol Res. 2017;43:265-71.

9. Li GT, Li XF, Wu B, Li G. Longitudinal parallel compression suture to control postopartum hemorrhage due to placenta previa and accrete. Taiwan J Obstet Gynecol. 2016;55:193-7.

10. Li GT, Li GR, Xu HM, Wu BP, Wang XN. Uterine folding hemostasis: a simpler and safer technique for controlling atonic postpartum hemorrhage. Arch Gynecol Obstet. 2016;294:689-95.

11. Farouk O, Elbasuony W, Elbohouty A. Uterine artery embolization versus surgical management in primary atonic postpartum hemorrhage: A randomized clinical trial. Egyptian Journal of Radiology and Nuclear Medicine. 2016;47:817-823

12. Matsubara S, Kuwata T, Baba Y, Usui R, Suzuki H, Takahashi H, et al. A novel 'uterine sandwich' for haemorrhage at caesarean section for placenta praevia. Aust N Z J Obstet Gynaecol. 2014;54:283-6.

13. El-Refaeey AA, Gibreel A, Fawzy M. Novel modification of B-Lynch uterine compression sutures for management of atonic postpartum hemorrhage: VV uterine compression sutures. J Obstet Gynaecol Res. 2014;40:387-91.

14. Mowat A, Minuzzo L, Wilson J. A necrotic uterus after a B-Lynch Suture: fertility sparing surgery. Aust N Z J Obstet Gynaecol. 2013;53:408-9.

15. Wang C, Mathur M. Severe distortion of the uterus associated with a B-Lynch suture. Int J Gynaecol Obstet. 2013;122:82-3.

16. Akbayir O, Corbacioglu Esmer A, Cilesiz Goksedef P, Ekiz A, Akca A, Guraslan B, et al. Single square hemostatic suture for postpartum hemorrhage secondary to uterine atony. Arch Gynecol Obstet. 2013;287:25-9.

17. Abdel-Aziz E, Reid B, C BL. A repeat B-Lynch suture for recurrent postpartum haemorrhage in a patient with factor V Leiden mutation. J Obstet Gynaecol. 2012;32:599-600.

18. Spatling L. "Quilting" sutures to prevent hysterectomy in patients with postpartum hemorrhage. Int J Gynaecol Obstet. 2012;117:291.

19. Lodhi W, Golara M, Karangaokar V, Yoong W. Uterine necrosis following application of combined uterine compression suture with intrauterine balloon tamponade. J Obstet Gynaecol. 2012;32:30-1.

20. Gezginç, K, Yazici F, Koyuncu T, Mahmoud AS. Bilateral uterine and ovarian artery ligation in addition to B-Lynch suture may be an alternative to hysterectomy for uterine atonic hemorrhage. Clin Exp Obstet Gynecol. 2012;39:168-70.

21. Alouini S, Coly S, Megier P, Lemaire B, Mesnard L, Desroches A. Multiple square sutures for postpartum hemorrhage: results and hysteroscopic assessment. Am J Obstet Gynecol. 2011;205:335 e1-6.

22. Poujade O, Grossetti A, Mougel L, Ceccaldi PF, Ducarme G, Luton D. Risk of synechiae following uterine compression sutures in the management of major postpartum haemorrhage. BJOG. 2011;118:433-9.

23. Zheng J, Xiong X, Ma Q, Zhang X, Li M. A new uterine compression suture for postpartum haemorrhage with atony. BJOG. 2011;118:370-4.

24. Matsubara S, Yano H, Taneichi A, Suzuki M. Uterine compression suture against impending recurrence of uterine inversion immediately after laparotomy repositioning. J Obstet Gynaecol Res. 2009;35:819-23.

25. Sentilhes L, Gromez A, Trichot C, Ricbourg-Schneider A, Descamps P, Marpeau L. Fertility after B-Lynch suture and stepwise uterine devascularization. Fertil Steril. 2009;91:934 e5-9.

26. Sentilhes L, Gromez A, Razzouk K, Resch B, Verspyck E, Marpeau L. B-Lynch suture for massive persistent postpartum hemorrhage following stepwise uterine devascularization. Acta Obstet Gynecol Scand. 2008;87:1020-6.

27. Nelson WL, O'Brien JM. The uterine sandwich for persistent uterine atony: combining the B-Lynch compression suture and an intrauterine Bakri balloon. Am J Obstet Gynecol. 2007;196:e9-10.

28. Harma M, Gungen N, Ozturk A. B-Lynch uterine compression suture for postpartum haemorrhage due to placenta praevia accreta. Aust N Z J Obstet Gynaecol. 2005;45:93-5.

29. Bhal K, Bhal N, Mulik V, Shankar L. The uterine compression suture--a valuable approach to control major haemorrhage at lower segment caesarean section. J Obstet Gynaecol. 2005;25:10-4.

30. Joshi VM, Shrivastava M. Partial ischemic necrosis of the uterus following a uterine brace compression suture. BJOG. 2004;111:279-80.

31. Hayman RG, Arulkumaran S, Steer PJ. Uterine compression sutures: surgical management of postpartum hemorrhage. Obstet Gynecol. 2002;99:502-6.

32. Shahin AY, Farghaly TA, Mohamed SA, Shokry M, Abd-El-Aal DE, Youssef MA. Bilateral uterine artery ligation plus B-Lynch procedure for atonic postpartum hemorrhage with placenta accreta. Int J Gynaecol Obstet. 2010;108:187-90.

33. Halder A. A new uterine suture technique to control PPH in congenitally malformed uterus during caesarean section. J Obstet Gynaecol. 2009;29:402-4.

34. Meydanli MM, Turkcuoglu I, Engin-Ustun Y, Ustun Y, Kafkasli A. Meydanli compression suture: new surgical procedure for postpartum hemorrhage due to uterine atony associated with abnormal placental adherence. J Obstet Gynaecol Res. 2008;34:964-70.

35. Hwu YM, Chen CP, Chen HS, Su TH. Parallel vertical compression sutures: a technique to control bleeding from placenta praevia or accreta during caesarean section. BJOG. 2005;112:1420-3.

36. Pal M, Biswas AK, Bhattacharya SM. B-Lynch Brace Suturing in primary post-partum hemorrhage during cesarean section. J Obstet Gynaecol Res. 2003;29(5):317-20.

37. Ochoa M, Allaire AD, Stitely ML. Pyometria after hemostatic square suture technique. Obstet Gynecol. 2002;99:506-9.

38. Cho JH, Jun HS, Lee CN. Hemostatic suturing technique for uterine bleeding during cesarean delivery. Obstet Gynecol. 2000;96:129-31.

39. Li GT, Li GR, Li XF, Wu BP. Funnel compression suture: a conservative procedure to control postpartum bleeding from the lower uterine segment. BJOG. 2016;123:1380-5.

40. Kaya B, Tuten A, Daglar K, Onkun M, Sucu S, Dogan A, et al. B-Lynch uterine compression sutures in the conservative surgical management of uterine atony. Arch Gynecol Obstet. 2015;291:1005-14.

41. Li GT, Li XF, Li J, Liu YJ, Xu HM. Reflexed Compression Suture for the Management of Atonic Postpartum Hemorrhage with an Abnormally Adherent Placenta. Gynecol Obstet Invest. 2015;80:228-33.

42. Li GT, Li XF, Liu YJ, Li W, Xu HM. Symbol "&" suture to control atonic postpartum hemorrhage with placenta previa accreta. Arch Gynecol Obstet. 2015;291:305-10.

43. Kaoiean S. Successful use of the B-Lynch uterine compression suture in treating intractable postpartum hemorrhage after cesarean delivery in Rajavithi Hospital. Journal of the Medical Association of Thailand. 2013;96:1408-1415

44. Penotti M, Vercellini P, Bolis G, Fedele L. Compressive suture of the lower uterine segment for the treatment of postpartum hemorrhage due to complete placenta previa: a preliminary study. Gynecol Obstet Invest. 2012;73:314-20.

45. Gottlieb AG, Pandipati S, Davis KM, Gibbs RS. Uterine necrosis: a complication of uterine compression sutures. Obstet Gynecol. 2008;112:429-31.

46. Wu HH, Yeh GP. Uterine cavity synechiae after hemostatic square suturing technique. Obstet Gynecol. 2005;105:1176-8.

47. Cotzias C, Girling J. Uterine compression suture without hysterotomy--why a non-absorbable suture should be avoided. J Obstet Gynaecol. 2005;25:150-2.

48. Huijgen QC, Gijsen AF, Hink E, Van Kesteren PJ. Cervical tourniquet in case of uncontrollable haemorrhage during caesarean section owing to a placenta accreta. BMJ Case Rep. 2013; doi: 10.1136/bcr-2013-009237.

49. Yoong W, Ridout A, Memtsa M, Stavroulis A, Aref-Adib M, Ramsay-Marcelle Z, et al. Application of uterine compression suture in association with intrauterine balloon tamponade ('uterine sandwich') for postpartum hemorrhage. Acta Obstet Gynecol Scand. 2012;91:147-51.

50. Ghosh SB, Mala YM. Alternate sequential suture tightening: a novel technique for uncontrolled postpartum hemorrhage. Obstet Gynecol Int. 2015;2015:145178.

51. Kaplanoglu M, Kaplanoglu DK, Koyuncu O. A different approach to placenta previa accreta: intrauterine gauze compress combined B-Lynch uterine compression suture. Clin Exp Obstet Gynecol. 2015;42:53-6.

52. Zhang ZW, Liu CY, Yu N, Guo W. Removable uterine compression sutures for postpartum haemorrhage. BJOG. 2015;122:429-33.

53. Kaplanoglu M. The uterine sandwich method for placenta previa accreta in mullerian anomaly: combining the B-lynch compression suture and an intrauterine gauze tampon. Case Rep Obstet Gynecol. 2013;2013:236069.

54. Canonico S, Arduini M, Epicoco G, Luzi G, Arena S, Clerici G, et al. Placenta Previa Percreta: A Case Report of Successful Management via Conservative Surgery. Case Rep Obstet Gynecol. 2013;2013:702067.

55. Huissoud C, Cortet M, Dubernard G, Tariel O, Fichez A, Escalon J, et al. A stitch in time: Layers of circular sutures can staunch postpartum hemorrhage. Am J Obstet Gynecol. 2012;206:177 e1-2.

56. Koyama E, Naruse K, Shigetomi H, Sado T, Oi H, Kobayashi H. Combination of B-Lynch brace suture and uterine artery embolization for atonic bleeding after cesarean section in a patient with placenta previa accreta. J Obstet Gynaecol Res. 2012;38:345-8.

57. Marasinghe JP, Condous G, Seneviratne HR, Marasinghe U. Modi fi ed anchored B-Lynch uterine compression suture for post partum bleeding with uterine atony. Acta Obstet Gynecol Scand. 2011;90:280-3.

58. Arduini M, Epicoco G, Clerici G, Bottaccioli E, Arena S, Affronti G. B-Lynch suture, intrauterine balloon, and endouterine hemostatic suture for the management of postpartum hemorrhage due to placenta previa accreta. Int J Gynaecol Obstet. 2010;108:191-3.

59. Marasinghe JP, Condous G. Uterine compression sutures for post-partum bleeding with atony; modification of the B-Lynch suture. Aust N Z J Obstet Gynaecol. 2009;49:67-70.

60. Dedes I, Ziogas V. Circular isthmic-cervical sutures can be an alternative method to control peripartum haemorrhage during caesarean section for placenta praevia accreta. Arch Gynecol Obstet. 2008;278:555-7.

61. Ouahba J, Piketty M, Huel C, Azarian M, Feraud O, Luton D, et al. Uterine compression sutures for postpartum bleeding with uterine atony. BJOG. 2007;114:619-22.

6. Ghezzi F, Cromi A, Uccella S, Raio L, Bolis P, Surbek D. The Hayman technique: a simple method to treat postpartum haemorrhage. BJOG. 2007;114:362-5.

63. Nelson GS, Birch C. Compression sutures for uterine atony and hemorrhage following cesarean delivery. Int J Gynaecol Obstet. 2006;92:248-50.

64. Habek D, Kulas T, Bobic-Vukovic M, Selthofer R, Vujic B, Ugljarevic M. Successful of the B-Lynch compression suture in the management of massive postpartum hemorrhage: case reports and review. Arch Gynecol Obstet. 2006;273:307-9.

65. Holtsema H, Nijland R, Huisman A, Dony J, van den Berg PP. The B-Lynch technique for postpartum haemorrhage: an option for every gynaecologist. Eur J Obstet Gynecol Reprod Biol. 2004;115:39-42.

66. Vijayasree M. Efficacy of Prophylactic B-Lynch Suture during Lower Segment Caesarian Section in High Risk Patients for Atonic Postpartum Haemorrhage. Kathmandu Univ Med J (KUMJ). 2016;14:9-12.

67. Sheikh S, Naz S, Shaikh A, Parveen R, Soomro N. B-Lynch suture in the management of massive post partum hemorrhage. Rawal Medical Journal. 2013;38:404-408

68. Ali MK, Badee AY, Abbas AM, Shazly SA. A novel technique for modified B-Lynch suture for the control of atonic postpartum haemorrhage. Aust N Z J Obstet Gynaecol. 2013;53:94-7.

69. Mostfa AA, Zaitoun MM. Safety pin suture for management of atonic postpartum hemorrhage. ISRN Obstet Gynecol. 2012;2012:405795.

70. Nanda S, Singhal SR. Hayman uterine compression stitch for arresting atonic postpartum hemorrhage: 5 years experience. Taiwan J Obstet Gynecol. 2011;50:179-81.

71. Somunkiran A, Ozdemir I, Demiraran Y, Yucel O. B-Lynch suture after the failure of hypogastric artery ligation to control post-partum hemorrhage due to placenta increta in a patient with the factor V Leiden mutation. J Obstet Gynaecol Res. 2007;33:557-60.

72. Api M, Api O, Yayla M. Fertility after B-Lynch suture and hypogastric artery ligation. Fertil Steril. 2005;84:509.

73. B-Lynch C, Coker A, Lawal AH, Abu J, Cowen MJ. The B-Lynch surgical technique for the control of massive postpartum haemorrhage: an alternative to hysterectomy? Five cases reported. Br J Obstet Gynaecol. 1997;104:372-5.

74. Aboulfalah A, Fakhir B, Ait Ben Kaddour Y, Asmouki H, Soummani A. A new removable uterine compression by a brace suture in the management of severe postpartum hemorrhage. Front Surg. 2014;1:43.

75. Stanojević D, Stanojević M, Zamurović M, Ćirović A, Hajrić A, Rakić S, Srbinović P. Uterine compression suture technique in the management of severe postpartum haemorrhage as an alternative to hysterectomy. Srpski Arhiv Za Celokupno Lekarstvo. 2009;137:638-640

76. Hillaby K, Ablett J, Cardozo L. Successful use of the B-Lynch brace suture in early pregnancy. J Obstet Gynaecol. 2004;24:841-2.
