# Supplementary material for: Systematic review on the needle and suture types for uterine compression sutures: a literature review
Source: BMC Surg. 2019 Dec 16;19:196. doi: 10.1186/s12893-019-0660-z (PMC6916157; doi:10.1186/s12893-019-0660-z)
Supplement: Supplementary file 3 — Additional file 3: Table S3 Comparison of uterine preservation rate among different suture sizes in uterine atony cases [file 12893_2019_660_MOESM3_ESM.docx]

| **Table S3** |  |  |  |  |  |  |
| --- | --- | --- | --- | --- | --- | --- |
| **Comparison of uterine preservation rate among different suture sizes in uterine atony cases** | | | | | | |
|  |  |  |  |  |  |  |
| Suture size | Suture material | Total cases | Mean blood loss (ml) | Transfusion rate^b^ | Uterine preservation rate | Severe complication rate^a^ |
|  |  |  |  |  |  |  |
|  | | | | | | |
| **No. 1** | All | 384 | 2167 | **211/372 (56.7%)^c^** | 360/384 (93.8%)^e^ | 5/384 (1.3%)^h^ |
|  | Polyglactin 910 and catgut | 309 | 2176 | **158/298 (53.0%)^d^** | 290/309 (93.9%)^f^ | 4/309(1.3%)^i^ |
|  | Limited transfusion cases (Polyglactin 910 and catgut) | | |  | 142/159 (89.3%)^g^ |  |
|  | | | | | | |
| **No. 2** | All | 279 | 2293 | **146/191 (76.4%)^c^** | 260/279 (93.2%)^e^ | 0/279(0%)^h^ |
|  | Polyglactin 910 and catgut | 254 | 2318 | **164/209 (78.4%)^d^** | 236/254 (92.9%)^f^ | 0/254(0%)^i^ |
|  | Limited transfusion cases (Polyglactin 910 and catgut) | | |  | 135/146 (92.5%)^g^ |  |

Bold indicates statistical significance.

^a^Severe complications are defined as need for surgical intervention to treat complications due to uterine compression sutures.

^b^The studies which did not mention the transfusion rate were excluded.

Statistical analysis was performed among c, d, e, f, g, h and i. The *p* values are listed below.

^c^*p* = 0.000.

^d^*p* = 0.000.

^e^*p* = 0.873.

^f^*p* = 0.733.

^g^*p* = 0.428.

^h^*p* = 0.077.

^i^*p* = 0.131.
